# A qualitative exploration of Pakistan’s street children, as a consequence of the poverty-disease cycle

**DOI:** 10.1186/2049-9957-3-11

**Published:** 2014-03-24

**Authors:** Muhammad Ahmed Abdullah, Zeeshan Basharat, Omairulhaq Lodhi, Muhammad Hisham Khan Wazir, Hameeda Tayyab Khan, Nargis Yousaf Sattar, Adnan Zahid

**Affiliations:** 1 Department of Community and Family Medicine, Shifa College of Medicine, Pitrus Bukhari Road, Sector H-8/4, Islamabad, Pakistan; 2 Department of Medicine, Shifa International Hospital, Pitrus Bukhari Road, Sector H-8/4, Islamabad, Pakistan; 3 Department of Medicine, District Headquarter Teaching Hospital, Rawalpindi, Pakistan

**Keywords:** Street children, Poverty-disease cycle, Pakistan

## Abstract

**Background:**

Street children are a global phenomenon, with an estimated population of around 150 million across the world. These children include those who work on the streets but retain their family contacts, and also those who practically live on the streets and have no or limited family contacts. In Pakistan, many children are forced to work on the streets due to health-related events occurring at home which require children to play a financially productive role from an early stage. An explanatory framework adapted from the poverty-disease cycle has been used to elaborate these findings.

**Methods:**

This study is a qualitative study, and involved 19 in-depth interviews and two key informant interviews, conducted in Rawalpindi, Pakistan, from February to May 2013. The data was audio taped and transcribed. Key themes were identified and built upon. The respondents were contacted through a gatekeeper ex-street child who was a member of the street children community.

**Results:**

We asked the children to describe their life stories. These stories led us to the finding that street children are always forced to attain altered social roles because health-related problems, poverty, and large family sizes leave them no choice but to enter the workforce and earn their way. We also gathered information regarding high-risk practices and increased risks of sexual and substance abuse, based on the street children’s increased exposure. These children face the issue of social exclusion because diseases and poverty push them into a life full of risks and hazards; a life which also confines their social role in the future.

**Conclusion:**

The street child community in Pakistan is on the rise. These children are excluded from mainstream society, and the absence of access to education and vocational skills reduces their future opportunities. Keeping in mind the implications of health-related events on these children, robust inter-sectoral interventions are required.

## Multilingual abstracts

Please see Additional file 1 for translations of the abstract into the six official working languages of the United Nations.

## Background

In his address at the launching ceremony of the Nelson Mandela Children’s Fund in 1995, Nelson Mandela said*: “There can be no keener revelation of a society’s soul than the way in which it treats its children.”*[1] This statement is often tested when it comes to the issue of street children. The term “street children” broadly refers to several groups: children on the streets, defined as those who maintain family ties and return home to sleep; and children of the streets, defined as those with limited family contact and who end up spending most of their days and nights on the streets [2].

Street children are a global phenomenon, with an estimated population of around 150 million across the world [3], but the actual numbers could be much higher. In 1989, UNICEF estimated 100 million children were growing up on urban streets around the world, and 14 years later reported that: “The latest estimates put the numbers of these children as high as 100 million”. Even in 2007 UNICEF said: “The exact number of street children is impossible to quantify, but the figure almost certainly runs into tens of millions across the world. It is likely that the numbers are increasing. [4]” Data on street children estimates that there is approximately 40 million street children in South America, 25 million in Asia, 10 million in Africa, and 25 million in Eastern and Western Europe [5]. While the largest concentrations of street children are reported in Latin and South America [2], South Asian countries have some of the highest numbers of street children in the world, with estimates ranging from around 18 million [6] in India to around 1.5 million in Pakistan [7].

The phenomenon of street children poses a multifarious societal challenge. In most cases, street children are the victims of poverty and factors closely associated with it [8]. Several other factors are usually considered to be responsible for the prevalence of street children, including conflicts within the family; poor parenting; physical, emotional, and sexual abuse; peer influence; domestic violence; death of parents; urbanization; famine; and war. All of these etiologies result in the need to seek opportunities outside of the home environment [9]. Street children in Pakistan perform a number of tasks ranging from begging, car washing, and scavenging, to pick pocketing and prostitution [10].

Pakistan is faced with the issue of street children like most other developing countries. A major reason for this constant influx of children on the streets is the rapid increase in the urban population and the inability of urban centers to accommodate them [11]. This has resulted in high rates of adult unemployment with a large number of families living on the edge of poverty. High population growth and smaller landholdings in the rural areas have led to high rates of urban migration to the cities already plagued with homelessness, child labor, and malnutrition [11].

In this paper, we discuss the issue of street children with reference to the vicious cycle of poverty and disease. It is a known fact that disease and poverty together form the perfect mix of circumstances for physically, socially, and financially deprived living conditions [12]. The public sector in Pakistan does not provide any long-term and sustainable forms of social or financial support to the poor. The constantly escalating numbers of children who are forced to work on the streets either to look after their households or simply to survive clearly demonstrate this [13]. This study purports to look into the issue of street children, with a focus on the circumstances that forced them to leave their homes and schools in order to generate income for survival. We discuss that, in the majority of the cases, there is evidence of a chronic disease at home that pushes these children to the streets.

## Methods

This was a qualitative study conducted in Rawalpindi from February to May 2013, on the streets and market places of Rawalpindi city, Punjab province, Pakistan. The main areas where the respondents came from were; Sadiqabad, Commercial Market (Satellite Town), and Chandni Chowk. The respondents for this study were selected according to the World Health Organization (WHO) definition of adolescents (10–19 years of age), working on the streets [14]. The sample size was 19 street children, selected using a non-random snowball sampling technique. A total of 31 children were approached and the study purpose was explained to them, in detail. Our sample size comprised the individuals who fully understood the purpose and gave verbal informed consent. Out of the adolescents selected for our study, 15 were working on the streets while the remaining four belonged to the group of children of the streets, who have limited or no family contact. When we started interviewing the respondents we felt that, based on similar circumstances, we achieved data saturation at 19 interviews. Other factors which governed this number were the resources that were available to us, as well as time constraints.

In addition, two in-depth interviews were conducted with key informants, who were adult men who had spent their childhoods working on the streets. The purpose of these interviews was to discuss the sensitive issues of sexual abuse and substance abuse, which were deemed inappropriate to discuss with the selected children respondents.

The data was collected by two teams of interviewers. Each team comprised of two interviewers and one supervisor. The teams were provided with transport and communication facilities. The team members received training on the interview guidelines. The research team comprised of two public health physicians, four medical students, a clinical psychologist, and a gatekeeper. Respondent were explained the purpose of the study and all their concerns were addressed. The confidentiality of all information was assured. Those who finally agreed to participate were asked to give verbal informed consent; the low literacy level of our study participants was acknowledged. The data was collected using an unstructured interview guide in the Urdu language to avoid language barriers. The main areas of focus in the data collection tool were demographic details of the street children, their family and social backgrounds, and the high-risk practices in which they are involved. Each interview took around 1–1.5 hours. The interviews were audio taped wherever permission was granted. The audiotapes were transcribed within four days of the initial interview. The tapes were destroyed 15 days after the interview. Key themes were identified and discussed after manual thematic content analysis was conducted.

### Ethical considerations

Ethical approval for this study was obtained from the Institutional Review Board Health Services Academy, Islamabad. Obtaining informed consent from children is a controversial subject, and when these children are unsupervised and without any guardians, such as in the case of street children, the vulnerability factor and ethical ambiguity rise even further. There are no set protocols regarding this issue, and international literature provides little clear guidance. Modern research ethics are based on principles which usually safeguard the interests of the participants, yet these laws do not signify the ultimate truth and, based on the research circumstances, are liable to change. The most significant ethical considerations that guided us through this study were beneficence and non-maleficence. The idea of the risk-benefit ratio and the intent of disseminating information about this vulnerable group guided us throughout the course of this study. We see these children everyday, yet we fail to play our part to bring them into a normal and socially productive life with ambitions and dreams. We felt that being researchers, the least we could do was discuss the problems faced by this group on an academic platform.

Informed consent was sought from the parents/guardians of 11 of the participants. The remaining eight consented after extensive explanation of the research process. For the children who had family contact, our first aim was to obtain verbal informed consent from a parent or caregiver. In cases where this was not possible, informed consent was sought from the participants themselves. Great care was taken to include only those children who consented and we took the information part of the informed consent very seriously. All children were repeatedly explained the purpose and methods of the study. Great care was taken to exclude those children who either failed to understand the purpose of the study or did not consent to the study.

## Results

The present study is a qualitative research paper, in which data was collected using in-depth interviews conducted with 19 children. We initially approached 31 individuals. Out of these, 19 gave informed consent after being clearly explained the purpose of the study.

### Basic demographics

The mean age of the respondents was 13.47 years ± 2.84 years; 14 were males while the remaining five were females. Out of the 19 respondents, six had exposure to formal schooling at the very basic levels, while 13 had never attended school. Most of the study participants had Pakhtoon ethnic origins (52%), followed by children with Punjabi backgrounds (21%), with the remaining children being of Afghan, Sindhi, or Saraiki descent. Average time spent on the streets was 7.18 years ± 4.71 years, and the average daily income was 2.92 USD/day ± 1.36 USD. Around 58% of the respondents had seven to 10 siblings (see Table 1).

**Table 1:** Basic demographics of the respondents

**Serial #**	**Age (years)**	**Gender (M/F)**	**Education**	**Ethnicity**	**Time living on the streets**	Average daily income PKR: (US$)*	**Type of work**	**Number of siblings**
1	10	F	Nil	Pakhtoon	3 years	300(4)	Begging	9
2	12	F	Nil	Pakhtoon	5 years	150(1.5)	Begging	12
3	18	M	4th grade	Punjabi	13 years	200(2)	Scavenging	7
4	11	M	Nil	Pakhtoon	2 years	200(2)	Begging, pick pocketing	8
5	14	M	2nd grade	Afghani	5 years	250(2.5)	Scavenging	9
6	17	M	Nil	Saraiki	13 years	300(3)	Balloon vender	4
7	19	M	Nil	Pakhtoon	16 years	500(5)	Scavenging, pick pocketing	14
8	15	M	Nil	Sindhi	10 years	300(3)	Begging	5
9	10	M	5th grade	Pakhtoon	6 months	350(3.5)	Begging	10
10	14	M	Nil	Punjabi	7 years	500(5)	Begging, selling flowers	7
11	12	F	Nil	Punjabi	8 years	250(2.5)	Begging	8
12	13	M	3rd grade	Punjabi	3 years	200(2)	Scavenging	10
13	17	M	Nil	Pakhtoon	14 years	200(2)	Selling flowers, scavenging	7
14	12	M	Nil	Pakhtoon	8 years	300(3)	Begging	5
15	11	F	Nil	Saraiki	2 years	100(1)	Begging	8
16	15	M	Nil	Punjabi	10 years	400(4)	Scavenging	6
17	15	M	Nil	Pakhtoon	10 years	600(6)	Pick pocketing, begging	6
18	11	F	1st grade	Pakhtoon	6 years	150(1.5)	Begging	4
19	10	M	4th grade	Pakhtoon	1 year	200(2)	Begging	7

*Average conversion rate 1 USD = 100PKR.

### Initiation of street life: the cage without bars

Most of the respondents had been exposed to life on the streets in their early years. The mean age of initiation of street life was 6.29 with a SD of ± 2.36. Most respondents maintained family contact and were pushed onto the streets due to financial circumstances. As one child said*: “I have been working on the streets since I was five years old. We are five siblings and our father passed away when I was three years old. Our eldest brother, who was 12 at the time, left school and started working as a dishwasher at a local restaurant. Later, as time passed and he alone couldn’t support us, I left school as well and started working with him”.* Life on the streets is tough and in most circumstances, it is governed by obligations rather than choices, as one respondent put it*: “No one wants to be in this situation. We want to go to school like other children, but going to school means that I would stop making money and will have to spend money on books and fees. I know many children who went to school, but are now working on the streets like me”.* Some of the participants also had social issues at home which overshadowed their financial conundrums: *“After my mother died, my father sent me to the city to work in a big house. The people in the house were nice but the other servants, especially their driver, used to beat me up a lot, so I ran away and went back to my father, who took me back because he said he had taken money from the people I worked for. I had no other option but to run away. Now I feel that I’m free as I can do whatever I want”.*

### Large families: more mouths to feed or more hands to work?

All of our respondents had large families, with a mean number of siblings 7.68 with SD ± 2.61. Pakistan has always had issues with family planning due to various socio-cultural barriers. People believe that having lots of children is somehow a sign of a successful life even if caring for these children gets out of control. During the course of this study, we met with a respondent’s mother who was begging on the streets alongside six of her children. She said: *“Rich people have lots of money while poor people have lots of children; this is what makes us rich”.* These large family sizes eventually have a toll on the children who are forced to drop out of schools and work on the streets. A study participant said*: “At first I was the only one out of my siblings working on the street selling balloons, while the remaining six went to school. But when my father became ill, one of my brothers and a sister had to join me. Now I sell balloons, while the younger two beg and sell flowers”.*

### Diseases at home: the poverty-disease cycle

The poverty-disease cycle clearly pointed to the flimsy equilibrium which exists between socio-economic statuses and the health of individuals, with the eventual burden materializing firstly on specific families, and then on communities in general.

Almost all of the study participants had some sort of health-related event in their families in the past that has affected their present and their future. As one respondent said: *“We used to have a happy life; all of my brothers and sisters used to go to school when our father was healthy, but since he fell ill, we have had to earn for the family. The two youngest siblings still go to school but the rest of us have to work. Maybe one day the younger ones will support us and we will not have to work on the streets”.* (See Table 2.)

**Table 2:** Health status of parents

**Serial #**	**Mortality status of parents**	**Current health status/Cause of death**
1.	Father dead, mother alive	Father died of tuberculosis, mother diabetic
2.	Both parents alive	Father drug addict, mother normal
3.	Father dead, mother alive	Father died of chronic liver disease (Hepatitis B/C?), mother Hepatitis C
4.	Both parents alive	Both parents normal
5.	Father alive, mother dead	Father drug addict, mother died due to pyrexia of unknown origin
6.	Unknown	Father had a leg injury on last contact, mother normal
7.	Both parents alive	Father normal, mother is asthmatic
8.	Both parents alive	Both parents normal
9.	Father dead, mother alive	Father died in a car accident, mother has arthritis and generalized weakness
10.	Both parents alive	Father has a spinal cord injury, mother normal
11.	Both parents dead	Father died of gunshot wounds, mother died of tuberculosis
12.	Both parents alive	Father drug addict, mother normal
13.	Both parents alive	Both parents normal
14.	Both parents alive	Father diabetic with left foot amputation, mother has some psychiatric problems
15.	Father alive, mother dead	Father normal, mother died of cancer
16.	Father dead, mother alive	Father died of acute respiratory problems, mother normal
17.	Both parents alive	Father had an episode of MI recently, mother has psychiatric complaints
18.	Father dead, mother alive	Father died in a road traffic accident, mother alive with painful joints leading to limited mobility
19.	Both parents alive	Father is a drug addict, mother normal

During the course of this study, it was observed that two basic ideologies originated as a result of experiencing the disruption of a normal family life due to various diseases involving the parents. Some respondents developed a fear of illness and saw a dark future based on their increased exposure. One child said: *“I want to live a long and healthy life with my family, but I know this is a just a dream. My father died because of tuberculosis when we were very young; in his last few days he often called me and told me important things. One day he told me to work hard and make lots of money because poor people die early. I have been trying to make money for many years, but finally I have realized that I will also die poor like my father because making money is a very difficult thing to do in our society”.*

Some study participants had seen their parents ill or dying, and as a consequence had developed a rebellious and carefree attitude. As one boy told us: *“I am not afraid of dying because every day is dangerous. Just last week, I got an electric shock from a live wire; it hurt a lot but look I am still alive. I cannot die till God wants me to, so why be afraid of anything? I do what I want and this is how I want to live”.*

When asked the about the reasons for working on the streets, all respondents enumerated a multitude of conditions at home, where various health-related events had forced them to work on the streets. These circumstances ranged from death to incapacitating diseases and parents’ substance addiction. One respondent said*: “Our father used to drive a van, but two years ago he had an accident. He is bedridden now and cannot work. My younger brother is sick most of the time and cannot go to school. The doctors say that his body doesn’t make fresh blood. Our mother has to stay home and take care of them; that is why she lost her job as a washing lady. Now I work in a dhabba (roadside restaurant). I make around 150–200 rupees (USD 1.5–2) per day to feed my family. How can we afford treatment for my father and brother; we are lucky to have some food every day”.*

### No education, no skills, no choices

Low literacy levels in developing countries when amalgamated with a lack of occupational opportunities create a complex situation where education alone has limited power over socio-economic tribulations. One of our respondents was of the opinion that: *“I learnt two very important things on the street: how to gather garbage and how to care for myself. Life is tough and you have to be smart to survive.”* Another participant told us that: *“I wish I had gone to school when I could. Now I know nothing besides blowing up and selling balloons. I don’t want to do this work forever. Do you think I can go to school at this age?”*

The importance of vocational skills is tremendous, as skills provide better occupational opportunities for earning a more stable livelihood. This was elaborated by one respondent who said: *“Just going to school won’t end my problems. I also have to make money for my family. I think schools should teach us how to do different things, instead of just teaching us how to read and write. This is why I stopped going to school”.*

### Push and pull factors

We asked our study respondents to tell us three things that they like and three things that they dislike about both street life, and life at home and school. Their responses are summarized in Table 3.

**Table 3:** Likes and dislikes regarding home and street life

	**Likes**	**Dislikes**
**Life at home**	1. Parents (mostly mothers)	1. Beatings from parents (mostly fathers)
	2. Availability of food	
	3. Shelter and clothing	2. Step parents
	4. Family structure	3. Not enough food
	5. Going to school	4. Too many people at home
	6. Pets at home	5. Too many problems
	7. Playing games with friends and siblings	6. Sick or impaired individuals present at home
	8. Free time	7. No freedom at home
	9. Watching television	
**Life on the streets**	1. Freedom to do anything	1. Rude people
	2. Adventure with friends	2. Police
	3. Ability to earn by myself	3. Fights between street children
	4. A lot of people know you	
	5. Have my own pet dog	4. Sleeping on the road side
	6. Get to eat anything I want	5. Bad people (sexual abuse)
		6. Some boys keep blades and hurt others during fights

### Sexual abuse and drug adictions in street children

We conducted two key informant interviews with adult men who were ex-street children, in order to gather information regarding the ethically sensitive issues of sexual abuse and drug addiction. Street children are more vulnerable to sexual exploitation because they are unsupervised. They are exposed to the dangers of the untamed urban culture of the developing world, where the poor implementation of laws, scarcity of resources, and meager literacy and awareness levels create the perfect recipe for child rights violations. Child molestation, nevertheless, cannot be attributed to a certain socio-economic or ethnic stratification. As one respondent told us: *“People who try to sexually abuse children do it mostly because they can. But the children on the streets have developed their own protective mechanisms; we look out for each other with the older ones in the groups keeping an eye on the younger ones. Once a boy from our group was kidnapped in a big white car. I noted the registration number and took the police to the address. We caught the man red-handed and the police took him away. He was a rich man and had no need to do something like this, but he did it because he thought he wouldn’t get caught. Just imagine how many children can’t get any help”.*

Substance abuse is another serious issue. Most children develop the habit of smoking at an early stage due to peer pressure or for acceptability. A common abusive substance is Samad Bond, a locally manufactured glue that is also used for volatile substance abuse. Our respondent told us that: *“Most kids sniff glue. Living on the streets is hard; sniffing glue makes you forget all your hardships. I used to sniff glue but when my friend got sick and died because of it, I stopped. Now I tell all the younger boys to stay away from glue sniffers, but only a few listen to me”.*

## Discussion

In most cases, poverty is a well-documented and plausible etiological feature in the initiation of street life of street children, along with an array of complex causes mostly prevalent in an urban environment [15]. Simultaneously, poverty itself does not occur in solitude and is further enhanced by diseases and their consequences. The poverty-disease cycle provides a clearer understanding of the issue, and how poverty originates and escalates in the presence of various debilitating illnesses [16].

We intend to take this academic debate further by linking the issue of street children with the poverty-disease cycle [15]. The circumstances that force children to spend their lives on the streets are always governed by obligations rather than choices [17]. Street children are not only deprived of their basic child rights but are additionally exposed to an array of hazards and risks that other children are protected from [18]. The socio-economic makeup of the homes that children leave behind to work on the streets is usually governed by poverty and disease [15], and this iterative relationship escalates with the passage of time based on the reciprocal liaison between these two factors.

**Figure 1:**
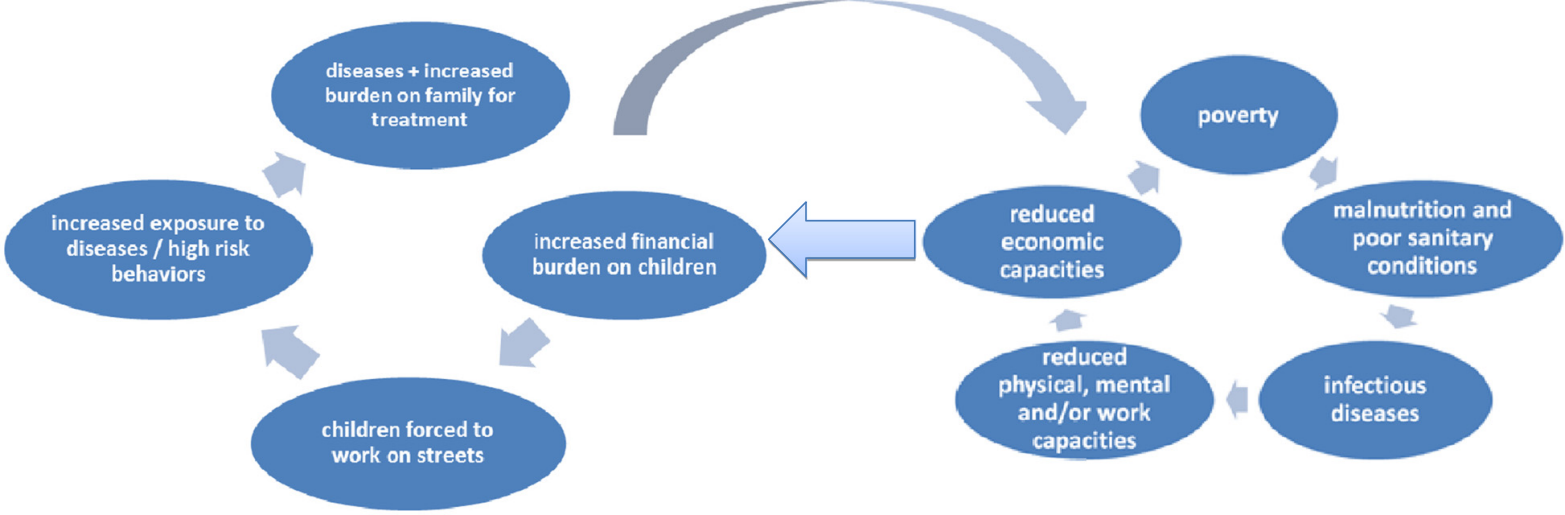
The relationship between poverty, disease and the phenomenon of street children.

In order to elaborate this further, we have developed a framework for explaining this association. In the light of our findings, we developed the idea that in most cases, if not all, the major factors that push children on the streets are governed my morbidity or mortality at home. The death or illness of family members have both short-term and long-term implications, and the financial onslaught of diseases force children to develop an income-generating role instead of simply enjoying their childhood [19]. (See Figure 1).

Another important aspect is the increased exposure to diseases of street children because of their altered social role. These children are exposed to infectious and non-infectious wastes during scavenging; they are also open to the elements of substance and sexual abuse [20]. Poor nutrition and exposure to environmental pollution also have long-term implications. Finally, violence in their daily life is a risk that always accompanies this ignored social group. Lack of financial, physical, and social accessibly to healthcare, as well as complicated health-seeking behaviors when added to the aforementioned problems, develop into a complex mix of circumstances which push these children further away from mainstream society [21].

## Conclusion

Street children are an everyday sight in almost all urban centers of the world, but in developing countries, the issue attains greater gravity based on the limited support these children get and the scarcity of resources. In Pakistan, where school enrolments are already poor, and poverty and illiteracy overshadow public law enforcement, the chances of children ending up on the streets increase exponentially. Health-related events almost always play a role. The current gaps between policy, planning, and implementation of interventions create many hurdles in the process of bringing about change for the marginalized communities. The efforts of the government and other organizations working for street children need to be organized under a single umbrella and informal education along with health, nutrition, and shelter services need to be structured for these less fortunate children so that they can have the opportunity to become productive members of society.

## Competing interests

The authors declare that they have no competing interests.

## Authors’ contributions

MA and ZB were the main authors of the manuscript and were involved in all aspects of the study. NY, HK, OL, HW, and AZ were involved in the development of the interview guidelines based on the original idea, as well as data collection and data analysis. OL and HW were involved in the data collection and analysis, and liaised with the gatekeeper. All co-authors have seen and approved the final version of the manuscript and have agreed to its submission for publication. All authors read and approved the final manuscript.

## Authors’ information

MA is a Senior Instructor at the Department of Community and Family Medicine at the Shifa College of Medicine. NY is an Instructor at the Department of Basic Health Sciences at the Shifa College of Medicine. ZB is pursuing his clinical internship at the Shifa International Hospital in Islamabad. HW, OL, and HK are fourth year MBBS students at the Shifa College of Medicine. AZ is a postgraduate trainee in medicine at the District Headquarters Teaching Hospital in Rawalpindi.

## Supplementary Material

Additional file 1 Multilingual abstracts in the six official working languages of the United Nations. Click here for file
