# Supplementary material for: A qualitative exploration of Pakistan’s street children, as a consequence of the poverty-disease cycle
Source: Infect Dis Poverty. 2014 Mar 24;3:11. doi: 10.1186/2049-9957-3-11 (PMC4022352; doi:10.1186/2049-9957-3-11)
Supplement: Additional file 1 — Multilingual abstracts in the six official working languages of the United Nations. [file 2049-9957-3-11-S1.pdf]

Translation of the abstract into the six official working languages of the United Nations

استكشاف نوعي لأطفال الشوارع في باكستان كنتيجة لدورة الفقر والمرض.

محمد أحمد عبد الله، زيشان بشارات، أميرول حق لودهي، محمد هشام خان وزير، حميدة طيب خان ، نرجس يوسف ستار، عدنان زاهد.

ملخص

خلفية: ان أطفال الشوارع ظاهرة عالمية ويقدر تعدادهم بنحو 150 مليون في جميع أنحاء العالم. ويشمل هؤلاء الأطفال العاملين في الشوارع ولكنهم على اتصال بعائلاتهم وأولائك الذين يعيشون في الشوارع بالفعل واتصالهم بعائلاتهم محدود أو منقطع. في باكستان، يضطر العديد من الأطفال إلى العمل في الشوارع بسبب أمور متعلقة بالصحة في بيوتهم والتي تتطلب من الأطفال القيام بدور تمويلي في مرحلة مبكرة من أعمارهم. وقد استخدم إطار تفسيري مقتبس من دورة المرض- و الفقر لشرح هذه الحقائق.

الطريقة: هذه الدراسة هي دراسة نوعية وتشمل 19 مقابلة طويلة ومقابلتين رئيستين وقد اجريت تلك المقابلات في روالبيندي – باكستان في الفترة من فبراير وحتى مايو 2013. وقد سجلت هذه البيانات ثم تم كتابتها. تم تحديد المواضيع الرئيسية ثم البناء عليها. تم الاتصال بهؤلاء الأطفال من خلال بواب كان من أطفال الشوارع سابقاً، من أفراد مجتمع أطفال الشوارع.

النتائج: طلبنا من الأطفال أن يصفوا حياتهم اليومية. أدت هذه القصص بنا إلى نتيجة مفادها أن أطفال الشوارع يجبرون دائما للعب أدوار اجتماعية متغيرة بسبب المشاكل المتعلقة بالصحة، والفقر، وحجم العائلة الكبيرة الذي لا يترك لهم اختيار سوى الدخول في سوق العمل وشق طريقهم. أيضاً جمعنا معلومات بشأن الممارسات ذات المخاطر العالية وزيادة مخاطر الاعتداء الجنسي والمخدرات، استنادا إلى زيادة تعرض أطفال الشوارع لهذه المخاطر. يواجه هؤلاء الأطفال قضية الاستبعاد الاجتماعي بسبب الأمراض والفقر ومن ثم يُدفعون إلى حياة مليئة بالمخاطر والأخطار؛ حياة تحصر دورهم الاجتماعي في المستقبل.

الاستنتاج: مجتمع أطفال الشوارع في باكستان في ازدياد. هؤلاء الأطفال مستبعدون من التيار المجتمعي الرئيسي. إن غياب فرصة الوصول للتعليم أو للمهارات الحرفية يقلل من فرصهم في المستقبل. مع الأخذ في الاعتبار الآثار المترتبة على الأحداث المتعلقة بالصحة على هؤلاء الأطفال. إن التدخل القوي من قبل القطاعات المعنية هام وضروري.

Translated from English version into Arabic by T. Catherine Hanna, through

巴基斯坦流浪儿童的定性研究：源于贫困和疾病循环链

Muhammad Ahmed Abdullah，Zeeshan Basharat，Omairul haq Lodhi， Muhammad Hisham Khan Wazir，Hameeda Tayyab Khan，Nargis Yousaf Sattar，Adnan Zahid

摘要

引言：流浪儿童是全球现象，估计全球大约有 1.5 亿流浪儿童。流浪儿童既包括那些与家庭保持联系但在街头谋生的儿童，也包括那些与家庭联系甚少或全无、完全以流浪街头为生的儿童。在巴基斯坦，很多儿童由于遭遇家庭的健康相关变故而不得不走上街头，过早的承担经济上的生产者角色。本研究应用适用于贫困-疾病循环链理论的释义框架来阐述相关研究的发现。

方法：该项研究是个定性研究，于 2013 年 2 月至 5 月在巴基斯坦拉瓦尔品第开展，内容涉及 19 个深度访谈和 2 个关键知情者访谈。对采集的数据首先进行录音再转化为相关材料。鉴别出关键主题并在其上进行深度挖掘。该研究通过一个看门人来接触受访者的，该看门人曾经也是一名流浪儿童，是这个群体的一员。

结果：我们要求这些流浪儿童描述自己的生活经历。从中我们发现，这些儿童都是由于健康问题、贫困或家庭过于庞大而不得不过早的自谋生计，从而转变成如今他们本不应该充当的社会角色。我们还发现，这些流浪儿童随着暴露于街头环境时间的增加，高危行为及性乱和物质成瘾等风险也在增加。疾病和贫困将这些儿童置于充满危险的生活环境之中，他们面临着被社会排斥的问题，而且这种生活也限制了他们未来的社会角色。

结论：巴基斯坦流浪儿童群体呈上升趋势，他们被排除在主流社会之外。教育和职业技能的缺乏减少了他们未来的机会。我们需要记住，发生在这些流浪儿童身上的健康相关变故，强有力的跨部门干预亟待开展。

Translated from English version into Chinese by Qian Men-bao, through

CHINESE CENTER FOR DISEASE CONTROL AND PREVENTION
NATIONAL INSTITUTE FOR PARASITIC DISEASES

Edited by Prof Zhou Xiao-nong (National Institute of Parasitic Diseases, Chinese Center for Disease Control and Prevention)

Une étude qualitative sur les enfants des rues au Pakistan, comme conséquence du cycle pauvreté-maladie

Muhammad Ahmed Abdullah，Zeeshan Basharat，Omairul haq Lodhi， Muhammad Hisham Khan Wazir，Hameeda Tayyab Khan，Nargis Yousaf Sattar，Adnan Zahid

Résumé

Vue d'ensemble : les enfants des rues constituent un phénomène mondial et on estime leur nombre à près de 150 millions. On compte parmi ces enfants ceux qui travaillent dans la rue tout en maintenant des liens avec leur famille, ainsi que ceux qui vivent pour ainsi dire dans la rue et n'ont que peu ou pas de contact avec leur famille. Au Pakistan, beaucoup d'enfants sont obligés de travailler dans la rue en raison des phénomènes d'altération de la santé qui surviennent à la maison et exigent que les enfants jouent un rôle productif sur le plan financier dès leur plus jeune âge. Un cadre explicatif adapté du cycle pauvreté-maladie a été utilisé pour aboutir à ces conclusions.

Méthodes : il s'agit d'une étude qualitative reposant sur 19 entretiens approfondis, réalisés à Rawalpindi, au Pakistan, entre février et mai 2013. Les entretiens ont été enregistrés sur bande audio et retranscrits. Des thèmes clés ont été identifiés et creusés. Les personnes interviewées ont été contactée par l'intermédiaire d'un ancien enfant des rues faisant partie de cette communauté.

Résultats : nous avons demandé aux enfants de raconter leur histoire personnelle. Ces témoignages nous ont permis de découvrir que les enfants des rues sont toujours obligés d'endosser un rôle social altéré, car les phénomènes d'altération de la santé, la pauvreté et le grand nombre d'enfants par famille ne leur laissent pas d'autre choix que d'entrer dans le monde du travail et de gagner leur vie. Nous avons également recueilli des informations sur les pratiques à haut risque et les risques accrus dus aux abus sexuels et à la consommation de drogues, en nous basant sur l'exposition accrue des enfants des rues. Ces enfants sont confrontés à l'exclusion sociale parce que les maladies et la pauvreté les conduisent à mener une vie remplie de risques et de dangers; une vie qui limite également leur rôle social pour l'avenir.

Conclusion : la communauté des enfants des rues au Pakistan est en augmentation. Ces enfants sont exclus de la société et l'absence d'accès à l'éducation et à la formation professionnelle réduit leurs perspectives d'avenir. De solides interventions

multisectorielles sont requises tout en gardant à l'esprit l'impact que peuvent avoir les phénomènes d'altération de la santé sur ces enfants.

Translated from English version into French by Stephbell, through

TRANSLATORS
WITHOUT BORDERS

Качественное исследование бездомных детей в Пакистане, как последствия цикла обусловленных бедностью заболеваний

Мухаммед Ахмед Абдулла, Зихан Башарат, Омаирул хак Лодхи, Мухаммад Хишам Хан Вазир, Хамида Тайяб Хан, Наргис Юсаф Саттар, Аднан Захид

Резюме

История вопроса: Бездомные дети — это международное явление, их численность по всему миру составляет порядка 150 миллионов человек. К данной категории относят детей, которые работают на улице, но поддерживают связь с семьей, а также тех, кто, по сути, живет на улице и не поддерживает или поддерживает ограниченную связь с родными. В Пакистане дети вынуждены работать на улице в связи с угрозой здоровью, с которой они сталкиваются дома. Это приводит к тому, что такие дети играют роль «добытчиков» с самого раннего возраста. Пояснительная структура, выработанная на основе цикла взаимозависимости бедности и заболеваний, использовалась для дальнейшей разработки полученных данных.

Методология: Данное исследование является качественным и включает 19 глубоких интервью, проведенных с двумя ключевыми респондентами в Равалпинди, Пакистан, в период с февраля по май 2013 года. Полученные данные были записаны на пленку, а затем транскрибированы. Были выявлены и получили развитие ключевые темы. Контакт с респондентами был налажен благодаря охраннику, который раньше также жил на улице и являлся членом уличного детского сообщества.

Результаты: Мы попросили детей рассказать о себе и своей жизни. Как оказалось, бездомные дети всегда вынуждены брать на себя измененные социальные роли в связи с проблемами со здоровьем, бедностью и большим размером семьи, в результате чего им не остается ничего другого, как начать работать и зарабатывать на жизнь самостоятельно. Кроме того, нам удалось собрать информацию в отношении видов деятельности, входящих в группу высокого риска, и повышенных рисков сексуального насилия и наркомании среди таких бездомных детей. Эти дети сталкиваются с проблемой социальной изоляции, так как болезни и бедность заставляют их сталкиваться с большим количеством рисков и опасностей, вести жизнь, которая ограничивает их социальную роль в будущем.

Заключение: Количество бездомных детей в Пакистане постоянно растет. Эти дети не являются членами основного общества, а отсутствие доступа к получению образования и профессиональных навыков сокращает их возможности в будущем. Принимая во внимание воздействие медицинских проблем и заболеваний на таких детей, необходимо принять ряд межсекторных мер.

Translated from English version into Russian by Irina Zayonchkovskaya, through

TRANSLATORS
WITHOUT BORDERS

Exploración cualitativa de los niños de la calle de Pakistán, como consecuencia del ciclo pobreza-enfermedad

Muhammad Ahmed Abdulla, Zeeshan Basharat, Omairul haq Lodhi, Muhammad Hisham Khan Wazir, Hameeda Tayyab Khan, Nargis Yousaf Sattar, Adnan Zahid

Extracto

Antecedentes: Los niños de la calle constituyen un fenómeno global, con una población estimada de alrededor de 150 millones en todo el mundo. Estos niños incluyen aquellos que trabajan en las calles pero conservan el contacto con la familia, y también aquellos que viven prácticamente en la calle y cuyo contacto con la familia es limitado o inexistente. En Pakistán, muchos niños se ven obligados a trabajar en las calles a causa de problemas relacionados con la salud en su hogar que fuerzan a los niños a desempeñar un papel económicamente productivo desde una edad temprana. Para desarrollar estos hallazgos se ha utilizado un marco exploratorio adaptado del ciclo pobreza-enfermedad.

Métodos: Este estudio es un estudio cualitativo consistente en 19 entrevistas en profundidad y dos entrevistas con informantes clave, realizadas en Rawalpindi, Pakistán, de febrero a mayo de 2013. Los datos se grabaron en audio y se transcribieron. Se identificaron y desarrollaron temas clave. Se contactó con los entrevistados por mediación de un asesor ex-niño de la calle que fue miembro de la comunidad de niños de la calle.

Resultados: Pedimos a los niños que nos relataran la historia de sus vidas. Estas historias nos llevaron al hallazgo de que los niños de la calle siempre se ven forzados a adoptar roles sociales alterados debido a que los problemas relacionados con la salud, la pobreza y el contexto de familia numerosa no les dejan más opción que incorporarse al mundo laboral para ganarse la vida. También obtuvimos información sobre las prácticas de alto riesgo y el mayor riesgo de abuso sexual y de abuso de sustancias, dependiente de la mayor exposición experimentada por los niños de la calle. Estos niños se enfrentan al problema de la exclusión social debido a que las enfermedades y la pobreza los empujan a una vida llena de riesgos y peligros, una vida que además restringe su rol social en el futuro.

Conclusión: La comunidad de niños de la calle de Pakistán está aumentando. Estos niños quedan marginados de la corriente dominante de la sociedad, y la falta de acceso a la educación y a la formación profesional reduce sus oportunidades futuras.

Teniendo presentes las implicaciones de los problemas relacionados con la salud en estos niños, es preciso poner en práctica sólidas intervenciones intersectoriales.

Translated from English version into Spanish by Elena de Terán Bleiberg, through

TRANSLATORS
WITHOUT BORDERS
